# Development of a virtual surgical plan for reverse shoulder arthroplasty as a treatment for complex proximal humerus fracture in an elderly patient

**DOI:** 10.1016/j.xrrt.2024.08.005

**Published:** 2024-08-30

**Authors:** Roelof J. van Luit, Jessie Rijntjes, Edsko Hekman, Lonneke Govaert, Freek Hollman, Femke F. Schröder, Egbert J.D. Veen

**Affiliations:** a Department of Orthopaedic Surgery, Medical Spectrum Twente, Enschede, The Netherlands; b Medical 3D Lab, Medical Spectrum Twente, Enschede, The Netherlands; c Technical Medicine, Faculty of Science and Technology, Technical Medical Centre, University of Twente, Enschede, The Netherlands; d Faculty of Engineering Technology, Department of Biomechanical Engineering, Technical Medical Centre, University of Twente, Enschede, The Netherlands; e Department of Orthopaedic Surgery, Viecuri Medical Centre, Venlo, The Netherlands

**Keywords:** Complex proximal humerus fractures, Virtual surgical planning, Computer assisted surgery, 3D technology, Tuberosity reattachment, Reverse shoulder arthroplasty

Proximal humerus fractures (PHFs) are the third most common fractures in the elderly.^3^ Often, they follow a complex fracture pattern with displaced tuberosities and impaired blood supply to the humeral head.^22^ For some patients with complex PHFs, a reverse shoulder arthroplasty (RSA) is indicated. Many studies show favourable outcomes of RSA compared to open reposition internal fixation and hemiarthroplasty in elderly patients with complex PHFs.^17^^,^^19^^,^^23^^,^^28^ During the surgical procedure, refixation of the tuberosities is paramount, as healing of the tuberosities is related to superior functional outcome.^4^^,^^13^^,^^20^ Currently, RSA for PHFs holds several difficulties. Firstly, the fracture pattern is identified on plain radiographs and computed tomography (CT), making it challenging to comprehend as bone fragments are usually displaced or rotated.^2^ Secondly, it is hard to determine and optimise the size and positioning of fragments and implants during surgery.^5^ These challenges might result in increased surgery time, risk of complications, and malreduction of fragments, leading to inferior outcome.^2^^,^^26^^,^^27^ With current commercially available arthroplasty-manufacturer-provided planning software, it is already possible to plan arthroplasty in the settings of fracture. However, it is not possible yet to manage and virtually reduce the fracture fragments. An upcoming technology to address this problem could be preoperative virtual surgical planning (VSP). This technique has already proven valuable in different types of surgery, such as in pelvic fractures^11^ and mandibular and maxillary reconstruction.^14^^,^^18^ Additionally, VSPs are already used in non-fracture shoulder arthroplasty with satisfying results.^16^^,^^25^ Therefore, it is hypothesised that a VSP could help illustrate the most favourable outcome of RSA for PHFs, thereby reducing complication risk and improving functional outcomes.

This study aims to develop, clinically implement, and evaluate a VSP for RSA in complex PHFs.

## Materials and methods

### Study design & patient characteristics

**Figure 1:**
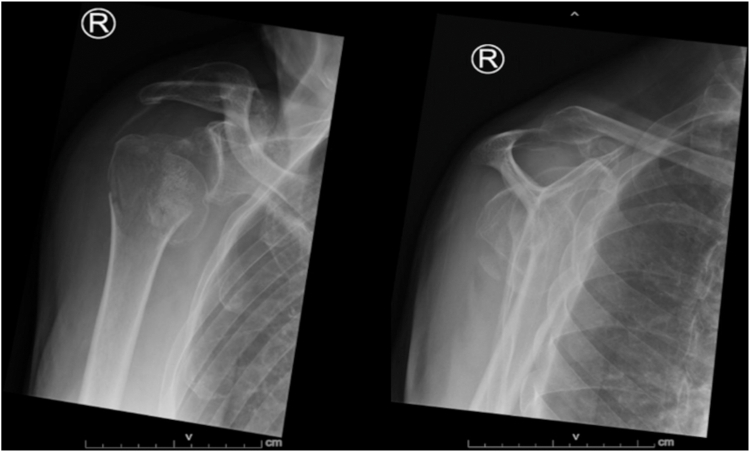
Anteroposterior (AP) (*left*) and lateral (*right*) preoperative plain radiographs of a complex four-part head split proximal humerus fracture. This case was used in the virtual surgical planning (VSP) for prospective use.

This proof of principle study contains two parts. Firstly, the VSP protocol is developed. For this, retrospectively four patients with a 4-part PHF, including fractured tuberosities that were refixated during RSA surgery between April 2021 and March 2022, were included. All patients (two male and two female) had left-sided PHFs. The mean age was 66 years (range: 60-76). Patient characteristics, plain radiographs, and CT scans were retrieved from the patients’ electronic health records. This also included images of the contralateral shoulder reconstructed from the initial CT scan. Secondly, the VSP was applied prospectively in one patient. For this, a 70-year-old male patient with a complex 4-part right-sided PHF after a fall from his bike was included (Fig. 1). He had no relevant medical history. The surgery took place fourteen days after the initial trauma.

The institutional review board of Medical Spectrum Twente approved the study (K22-24). Retrospectively included patients were exempted from informed consent. Written informed consent was obtained from the prospective included patient.

### VSP development

For all patients, the CT and plain radiographs were anonymised and imported into the 3-dimensional (3D) analysis software Materialise Mimics, version 24.0 (Leuven, Belgium). To virtually plan the prosthesis, CT scans of separate prosthesis parts and sizes were acquired (Comprehensive system; Zimmer Biomet, Warsaw, IN, USA).

**Figure 2:**
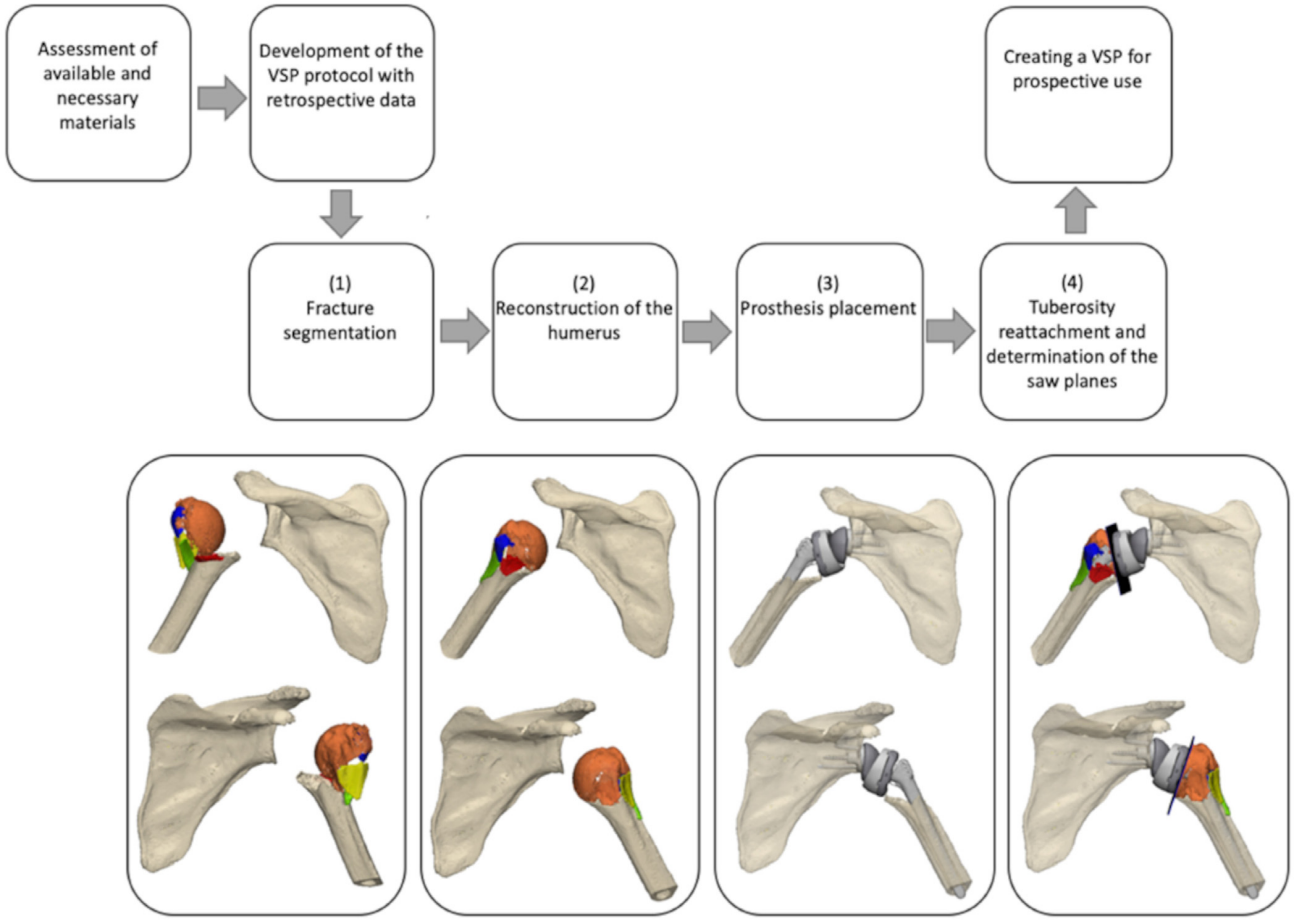
Step-by-step guide. Developing a VSP for complex proximal humerus fractures (PHFs) in four steps. 1) Fracture segmentation, 2) humerus reconstruction, 3) prosthesis placement, and 4) reattachment of the tuberosities and determining the saw plane. *VSP*, virtual surgical planning.

Preoperative CT scans of both shoulders and separate implant parts were used. Next, the VSP was developed using the following steps (Fig. 2). 1: Using the anonymised data of the four retrospective patients, the fracture pattern was semiautomatically segmented based on thresholding in Materialise Mimics. 2: The normal anatomy of the fractured shoulder was reconstructed by translating and rotating the different segmented fracture parts using a mirrored scan of the non-affected shoulder with the knowledge of anatomic landmarks using 3D medical image processing software (3-Matic 17.0; Materialise, Leuven, Belgium). 3: The optimal position and size of the fracture stem and baseplate glenosphere, including the position of bone cuts, were determined, and the prosthesis was virtually implanted. 4: The fragments containing the tuberosities were identified and placed onto the humeral shaft (Video). Additionally, autologous bone grafts could be used if the tuberosity fragments were too small. In this case, this was not necessary. To optimize the VSP protocol, the VSPs of the retrospective cases were compared to the postoperative radiographs and discussed with the orthopedic surgeon (E.J.D.V.).

### Prospective case

For the prospective case, the above-described methodology was used to create a VSP based on the preoperative CT scan, in consultation with the surgeon. The RSA was implanted in a beach chair position using a standard deltopectoral approach. A standard inferior baseplate position with slight anteversion was employed to create overhang and prevent notching. A common configuration of five nonresorbable sutures (FibreWire; Arthrex, FL, USA) were passed through the diaphysis, fragments, and stem. After cementing the stem in the planned height according to the VSP, the fragments were reduced and secured. Intraoperatively, the VSP was shown on the monitors which were particularly helpful during the sizing of the fragments, implant height, and reduction of fragments. The patient could be discharged the next day in good health and started with guided physical therapy.

### Outcomes

**Figure 3:**
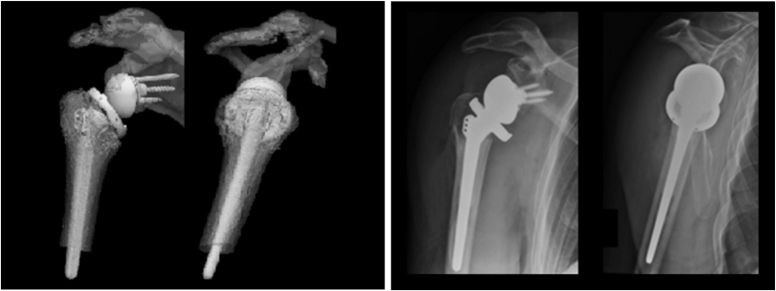
Postoperative AP and lateral views of the virtually planned a reverse shoulder arthroplasty (*left*) and postoperative radiographs (*right*), showing good position and size of the implant and anatomic reduction of the tuberosities. *AP*, anteroposterior.

The VSP for prospective use was evaluated by the surgeon with a questionnaire directly after the surgical procedure. This questionnaire consisted of ten questions designed to assess the additional value of the VSP protocol according to the orthopedic surgeon. The position of the RSA was assessed on postoperative plain radiographs and reconstructed anteroposterior and lateral views from the VSP, based on adequate reduction of fracture fragments, prosthesis position and size (Fig. 3). The Oxford Shoulder Score (OSS) was performed at both two and six months to assess surgical outcome, patient satisfaction, functional outcome, and pain.^6^

## Results

**Figure 4:**
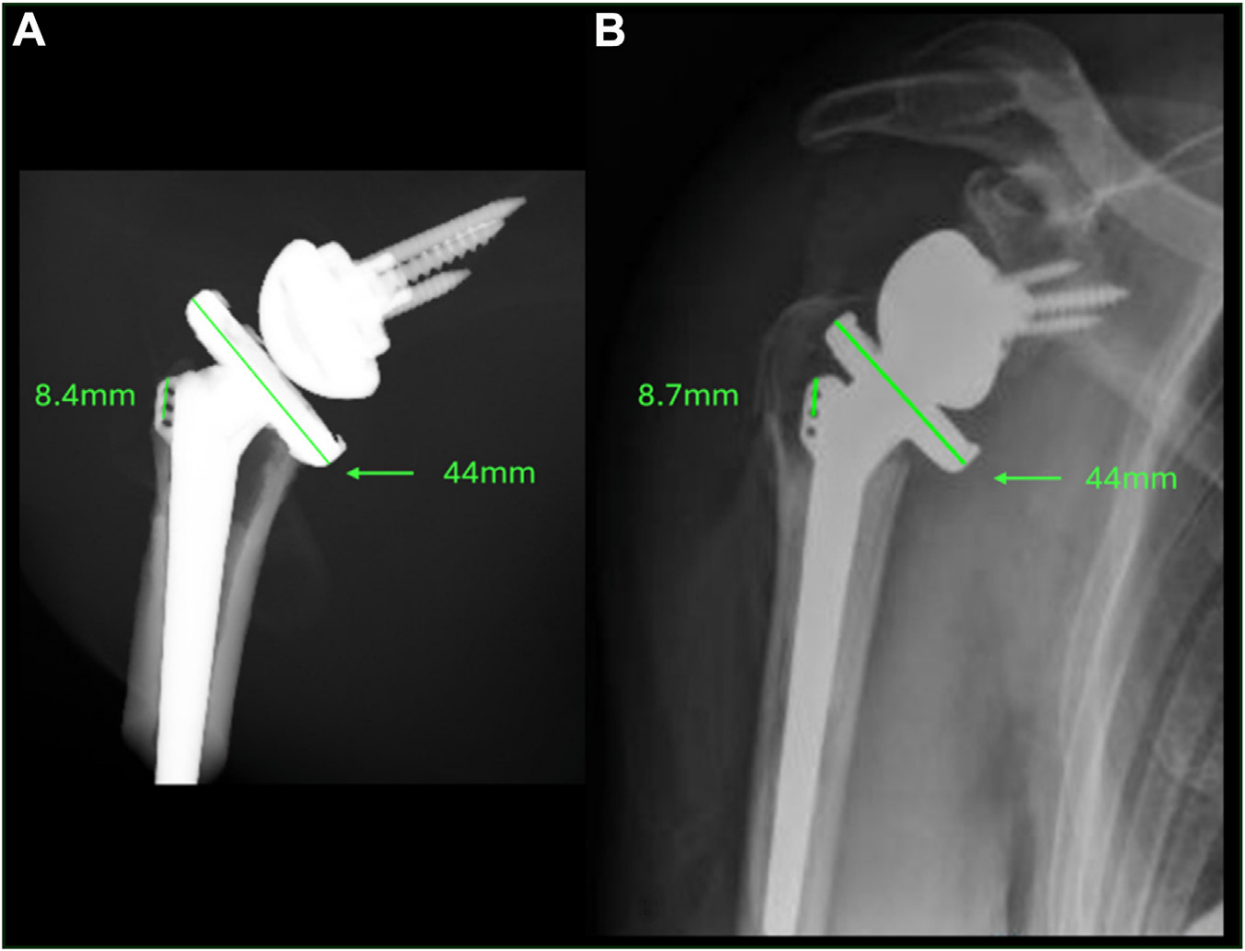
(**A**) The digitally reconstructed radiograph created using the VSP at the same angle as the postoperative radiograph. (**B**) The postoperative radiograph in AP direction, calibrated for measurements using the size of the prosthesis (*right*). The humeral tray was used for calibration (44 mm). The difference in planned and realized stem height is 0.3 mm (measured from proximal stem suture holes to medial calcar plane). *AP*, anteroposterior; *VSP*, virtual surgical planning.

The designed methodology was implemented with satisfying results in a prospective case. According to the orthopedic surgeon, (EJDV), the VSP (Fig. 2) gave helpful insights into the fracture pattern, fragment size, and tuberosity repositioning, resulting in improved preparation and more confidence during the surgery. The postoperative plain radiographs showed good position, adequate sizing of the implants, and reduction of the tuberosities, comparable to the reconstructed anteroposterior and lateral view from Materialise Mimics (Fig. 3). The planned and definite stem heights were compared by a digital reconstruction radiograph using the VSP at the same angle as the postoperative radiograph. The magnification was calibrated on the size of the prosthesis. The difference in stem height was 0.3 mm (Fig. 4).

During the postoperative appointments, the patient was asked to evaluate the treatment using the OSS. He reported excellent outcomes (score 48/48) two and six months after surgery.

## Discussion

In this study, a method for VSP for complex PHFs in the elderly patient was successfully developed using retrospective data from four patients and subsequently implemented and evaluated in one prospective case. The VSP procedure was evaluated using an expert evaluation, and the outcome was evaluated by assessing plain radiographs and the OSS after two and six months.

To the best of our knowledge, this is the first study to develop a VSP for the reattachment of tuberosities to a RSA. Therefore, the result cannot be compared to similar studies. However, multiple studies have investigated the additional value of 3D planning open reposition internal fixation procedures for complex PHFs.^27^^,^^30^ It is stated that 3D printing and online 3D segmentation of the injury site provide helpful information about the injury and the size of the bony fragments.^11^^,^^21^ In systematic reviews, Lal *et al* and Tack *et al* showed that 3D planning resulted in reduced surgery time, blood loss, and intraoperative fluoroscopy in a broad spectrum of procedures in the field of orthopedic, neurosurgical, maxillofacial, and trauma surgery.^15^^,^^24^ In line with the results of these systematic reviews, we expect similar beneficial results from our new VSP method for complex PHFs.

Besides assisting the orthopedic surgeon in planning the reduction of the tuberosities, another important advantage of this VSP lies in the resection plane of the humerus head, determining stem height. This plane is not specifically described in the literature and can be challenging to determine during surgery by complex PHFs since normal anatomy is disturbed. Cagle *et al* addressed this problem before and recommended using 5 cm between the superior border of the pectoralis tendon and the most superior aspect of the metallic humerus component as a resection angle to approximate the ideal plane.^5^ However, this only approximates the optimal stem height. An advantage of using our VSP method is that the contralateral nonaffected shoulder can be mirrored and used as a target for reconstruction, which made it possible to exactly preplan the humeral head resection plane, stem size, and height. Furthermore, no additional imaging and, thereby unnecessary radiation exposure to the patient is required to obtain data on the contralateral shoulder since the contralateral shoulder already is within the field of view of the initial shoulder CT. Nonetheless, planning might be difficult if no scan of the contralateral shoulder is obtained initially, or when the patient has an abnormal anatomy of the contralateral shoulder

A limitation of using a VSP for complex PHFs is that it is only applicable in hospitals collaborating with a medical 3D lab or having the knowledge of and access to 3D planning software. Furthermore, the applicability of the reconstruction protocol highly depends on the fracture pattern. Virtual reconstruction was relatively uncomplicated in this case since the fracture consisted of five larger fragments, and anatomic landmarks were recognizable. Also, the soft tissue tension in this case was relatively low since there was no preoperative stiffness and the time from injury to surgery was relatively short (fourteen days). Reconstruction can be more challenging in patients with more complex fractures and increased soft tissue tension.

According to the orthopedic surgeon, a point of improvement was the possibility of segmentation of the trabecular bone and providing accurate measurements of bone fragment size. However, this is challenging to improve since CT scans provide relatively little information about trabecular bone and soft tissue due to their low value in hounsfield units. An additional CT analysis, as demonstrated by Arenas-Miquelez et al, showed that segmentation of the rotator cuff muscles can be achieved with CT data.^1^ Yet, the use of this technique might be limited in the presence of fracture haematoma. Furthermore, segmentation of these structures is possible in Materialise Mimics with magnetic resonance imaging (MRI) data.^9^ MRI or CT fusion scans are already successfully being applied in the fields of maxillofacial, cranial, and spinal surgery.^7^^,^^8^^,^^10^^,^^14^^,^^29^ The significance of a fused CT or MRI for visualizing soft tissue and trabecular bone in a VSP is unclear yet. We hypothesize that this can further improve the understanding of the fracture pattern and might be used as a predictor for postoperative mobility.

Lastly, to maximize the benefits of the VSP during surgery, mixed reality technology could be an interesting addition in the future. Mixed reality technology is an upcoming new method to improve anatomical understanding and bridge the gap between preoperative planning and surgical execution using 3D holograms of the anatomy and VSP.^12^

This proof of principle study shows promising results in one prospective case. In the future, we aim to implement this VSP into our clinical practice for complex PHFs. Therefore, in the coming research, we hope to assess the additional value of the VSP in terms of functional outcomes for the patient. We expect that the VSP will improve the patient's functional outcomes by a more optimal reduction and fixation of the tuberosities and the height of the humeral stem and by reducing surgery time and, thereby, also the risk of complications.

## Conclusions

In this proof of principle study, a VSP for RSA in complex PHFs was successfully developed using retrospective data, implemented prospectively in a case study, and evaluated. The VSP was valuable to the orthopedic surgeon by providing information about the fracture complexity, resection planes, and prosthesis placement. The patient showed satisfactory radiological and clinical outcomes for two and six months postoperatively. Future research should focus on assessing the additional value of the VSP in terms of improving the procedure (surgery time, blood loss, and the use of intraoperative fluoroscopy) and outcomes for the patient (pain, range of motion, and complications).

## Disclaimers:

Funding: No funding was disclosed by the authors.

Conflicts of interest: The authors, their immediate families, and any research foundation with which they are affiliated have not received any financial payments or other benefits from any commercial entity related to the subject of this article.
